# A serious adverse surgical event: Management of suspected HSV-1 keratitis in a donor cornea

**DOI:** 10.1007/s00717-016-0325-6

**Published:** 2017-01-30

**Authors:** Rebecca Kaye, Bernhard Steger, Jern Y. Chen, Vito Romano

**Affiliations:** 1 Department of Corneal and External Eye Diseases, St. Paul’s Eye Unit, Royal Liverpool University Hospital, Liverpool, UK; 2 Department of Eye and Vision Science, University of Liverpool, Liverpool, UK; 3 Department of Eye and Vision Science, University Hospital Southampton, Tremona Road, SO16 6YD Southampton, UK

**Keywords:** Herpes Simplex (HSV), Keratitis, Donor, Recipient, Corneal Transplant, Herpes Simplex (HSV), Keratitis, Spender, Empfänger, Hornhauttransplantation

## Abstract

**Purpose:**

To describe the management of a serious adverse event in a patient undergoing penetrating keratoplasty (PK).

**Case report:**

A 68-year-old man underwent PK for an aphakic bullous keratopathy following previous complicated cataract surgery. He had no past history of herpetic disease. Storage of the corneoscleral disc in the transport bottle precluded microscopic examination. After placement of the trephined donor cornea on the open eye of the recipient, a large dendritiform geographic ulcer was noted on the donor cornea. A replacement cornea was used after changing potentially contaminated instruments. Intravenous antiviral treatment was commenced intraoperatively to reduce the risk of infection to the central nervous system. Postoperatively, oral and topical antiviral treatment was commenced and 6 months following surgery the patient developed a geographic corneal ulcer at the graft host interface.

**Conclusion:**

Containers to transport corneoscleral discs should enable microscopic examination by the surgeon prior to use. High dose systemic antivirals may reduce the risk of herpetic disease involving the posterior segment of the eye and neuroretina in the aphakic eye and spread to the central nervous system.

## Zusammenfassung

**Zweck:**

Beschreibung des Managements eines unerwünschten Ereignisses bei einem Patienten, der eine penetrierende Keratoplastik (PK) erhielt.

**Fallbericht:**

Ein 68-jähriger männlicher Patient unterzog sich einer PK aufgrund einer aphaken bullösen Keropathie nach einer früheren komplizierten Kataraktoperation. Er hatte keine Vorgeschichte einer Herpeserkrankung. Aufgrund der Aufbewahrung der korneoskleralen Scheibe in der Transportflasche war eine mikroskopische Untersuchung nicht möglich. Nach dem Einbringen der präparierten Spenderhornhaut in das offene Auge des Empfängers wurde ein großes dendritisches (geographisches) Ulkus auf der Spenderhornhaut festgestellt. Es wurde eine Ersatzhornhaut verwendet, nachdem die potenziell kontaminierten Instrumente ausgetauscht worden waren. Intraoperativ wurde eine intravenöse antivirale Behandlung begonnen, um das Risiko einer Infektion des Zentralnervensystems zu reduzieren. Postoperativ wurde mit der oralen und topischen antiviralen Therapie begonnen. Sechs Monate nach der Operation entwickelte der Patient ein geographisches Hornhautulkus an der Grenzfläche von Transplantat und Wirtsgewebe.

**Schlussfolgerung:**

Behältnisse zum Transport von korneoskleralen Scheiben sollten vor deren Verwendung eine mikroskopische Untersuchung durch den Operateur erlauben. Hoch dosierte systemische antivirale Medikamente könnten das Risiko einer herpetischen Erkrankung reduzieren, die das hintere Augensegment sowie die Neuroretina im phakischen Auge betreffen und eine Ausbreitung auf das Zentralnervensystem nach sich ziehen kann.

## Introduction

Herpes simplex keratitis (HSK) is one of the major causes of corneal inflammation and corneal blindness, rated just second to trauma in most developed countries [1–3]. Clinical reports support the possible transmission of herpes simplex virus type 1 (HSV-1) via donor corneas and its role in graft failure [4]. Cleator et al. reported a case of primary graft failure due to presumed donor-to-host transmission of HSV following organ culture in a patient who underwent a penetrating keratoplasty for keratoconus [5]. It has also been reported that the HSV-1 may remain viable in stored corneal tissue at 4° [6]. There are reports of post-keratoplasty HSV-1 infection in patients with no history of an herpetic infection [7, 8] and evidence of donor-to-host transmission of the virus has also been demonstrated in animal studies. In rabbits, latently infected donor tissue can induce HSV-1 infection in naive recipients after penetrating keratoplasty [9]. HSV-1 inoculation in the anterior chamber of one eye has been shown to lead to the spread of virus to the brain, contralateral optic nerve and then posterior segment of contralateral eye [10, 11]. We report a case of clinically suspected HSK in the donor cornea following organ culture and subsequently in the recipient following penetrating keratoplasty and the perioperative management to reduce the infection risk and development of disease of the central nervous system.

## Case report

**Fig. 1:**
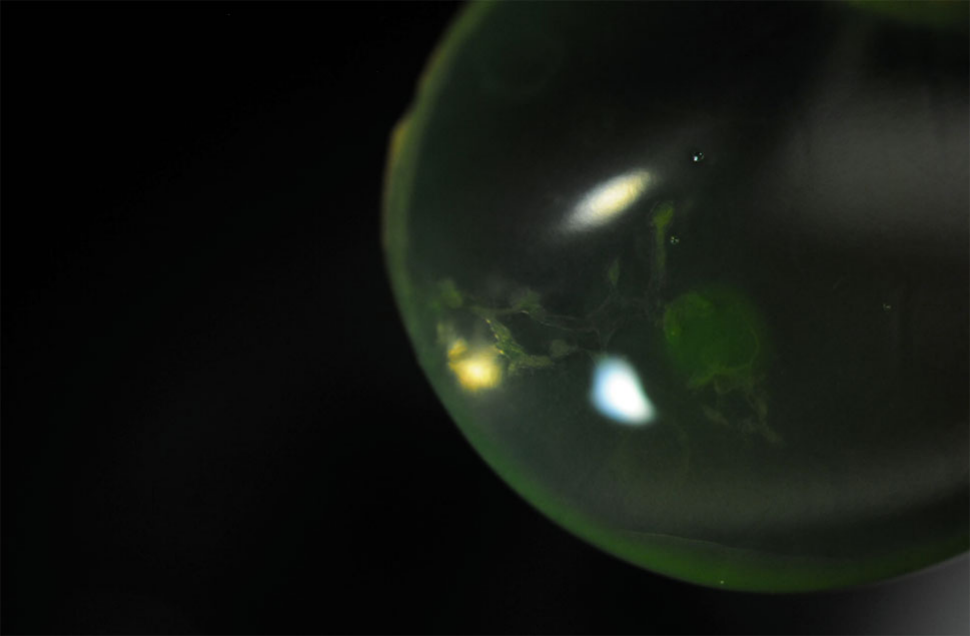
Dendritic-geographic epithelial ulcer on the donor tissue

A 68-year-old man underwent penetrating keratoplasty for aphakic bullous keratopathy and corneal scarring. He had no history of herpetic disease. The donor cornea originated from a 71-year-old female who died due to chronic pulmonary disease. The time interval between death and transfer of the corneoscleral button into organ culture medium was 36 h. The endothelial count was 2400cell/mm^2^ with mild polymegethism and pleomorphism. A 8.5 mm Hessberg–Barron trephine was used to prepare the donor cornea and a 8 mm trephine for the recipient. After placement of the donor onto the aphakic eye a large dendritic-geographic epithelial ulcer was noted (Fig. 1). The graft was removed, all surgical instruments were changed and the surgical staff regowned. Fortunately, a second donor corneoscleral disc was available, which was then used to complete the operation instead of resuturing the patient’s own cornea back into place. The second donor originated from a 69-year-old male who died from a myocardial infarction. The time interval between death and transfer of the corneoscleral button into organ culture medium was 40 h. The endothelial count was 2600cell/mm^2^ with some variation in cell size and shape.

**Fig. 2:**
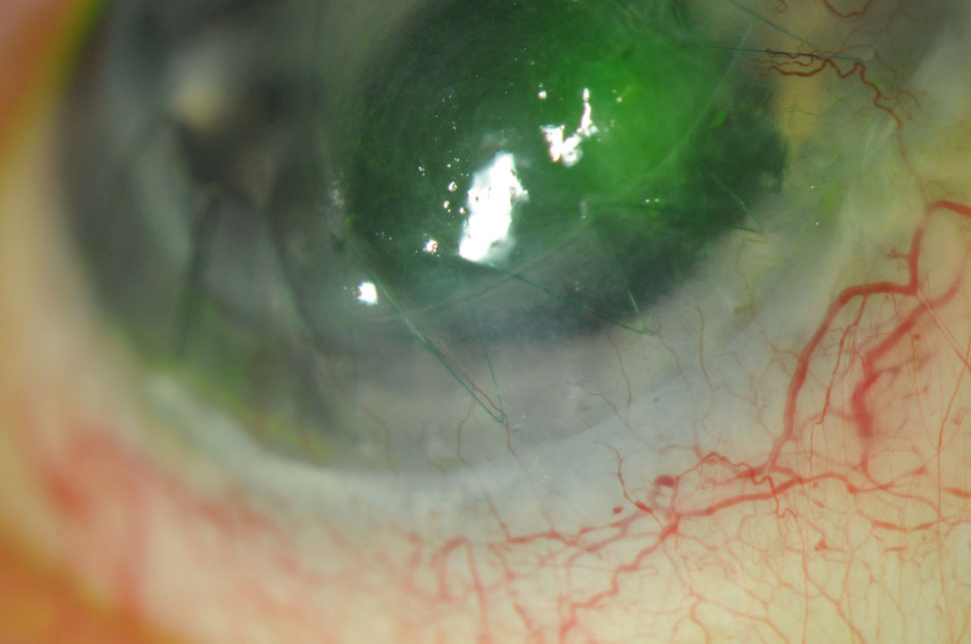
Geographic corneal ulcer involving the graft-host interface

Perioperative intravenous aciclovir was administered intravenously (5 mg/kg). Postoperatively, the patient was commenced on systemic and local antiviral therapy for 1 week (valaciclovir 1 g three times a day, acyclovir ointment five times per day) and local antibiotic treatment (chloramphenicol four times a day). Topical steroids were withheld for 48 h. At 1 week the graft was clear and had re-epithelialized. Long term prophylactic acyclovir 400 mg twice a day was commenced but 6 months later the patient developed a geographic corneal ulcer involving the graft-host interface (Fig. 2) with keratic precipitates and corneal oedema (central corneal thickness 710 μm). The patient was treated with acyclovir 400 mg and topical aciclovir both five times a day for 2 weeks with resolution of the ulcer and residual scarring. Although the patient had HSV IgG serum antibodies, evidence of HSV-1 DNA was not detected in the first donor cornea or following sampling of the corneal ulcer via PCR and culture methods.

## Discussion

The potential transmission of HSV-1 from donor to host has been recognized by clinical and laboratory studies [12–14]. Viral particles have been found in keratocytes and endothelial cells [15] of corneal grafts with postoperative keratitis. HSV-1 is known to induce endothelial cell necrosis during organ culture, consistent with reports of postoperative keratitis [5, 16]. A rate of 1.2 per 1000 person-years has been reported for newly acquired herpetic keratitis in the early postoperative period [12]. Borderie et al. reported that HSV-1 was responsible for 3 out of 4 primary graft failures among 586 consecutive grafts performed [17]. Evidence of HSV-1 has been found in 10% of patients with diseased corneas with no history of HSK undergoing corneal transplantation [16] and also in cell pellets obtained from donor cornea culture medium in (%) (3 out of 80) donor corneas [18, 19]. Currently, however, screening assays are not undertaken by eye banks to identify HSV-1 in the donor cornea or medium during storage.

We describe a case of clinically suspected HSK in the donor cornea and subsequently in the recipient. Biswas et al. [20] described the development of primary graft failure and ulcerative keratitis in two patients, respectively [20]. Similar to our patient, they suggested transmission of HSV-1 through organ-cultured corneal graft in two patients in spite of negative results of PCR performed on both recipient corneas, based on the presence of endothelial cell necrosis in the fellow donor cornea in one of the cases. In the presented case HSV-1 originating from the host cannot be ruled out as the source of the described viral keratitis, but is deemed highly unlikely given the absence of previous herpetic disease. Although we were not able to isolate HSV-1 either from the donor or host, the clinical appearances were typical in both the donor and host. Intraoperative and perioperative antiviral treatment may have prevented potential intraocular spread to the posterior segment as described by Atherton and Streilein [10, 11] following anterior chamber inoculation and subsequent disease of the central nervous system in a similar mechanism to oronasal and vomeronasal spread [21–23]. This case highlights the issue of being able to adequately examine the donor corneoscleral disc prior to use. Adequate biomicroscopic visualization of the donor cornea is not possible in the storage transport containers currently used for organ cultured corneas. This problem could be addressed by redesigning the transport chamber used for organ cultured corneas, or the use of hypothermic transport containers, which allow assessment of donor tissue by slit lamp biomicroscopy or the operating microscope before use [24].
